# Comparison of clinicopathological features and prognosis of papillary thyroid carcinoma and microcarcinoma: A population-based propensity score matching analysis

**DOI:** 10.3389/fendo.2022.944758

**Published:** 2022-08-05

**Authors:** Bei Qian, Longqing Hu, Shoupeng Zhang, Junlin Zhu, Li Mei, Tao Huang, Xincai Qu

**Affiliations:** Department of Thyroid and Breast Surgery, Union Hospital, Tongji Medical College, Huazhong University of Science and Technology, Wuhan, China

**Keywords:** papillary thyroid carcinoma (PTC), papillary thyroid microcarcinoma, (PTMC), propensity score matching (PSM), clinicopathological features, active surveillance (AS)

## Abstract

**Background:**

Overtreatment of papillary thyroid microcarcinoma (PTMC) has become a common concern. This study aimed to compare clinicopathological features between PTMC and papillary thyroid carcinoma (PTC) and to explore whether surgery can confer significant survival benefits in all patients with PTC or PTMC.

**Methods:**

Data of 145,951 patients with PTC registered in Surveillance, Epidemiology, and End Results (SEER) database and 8,751 patients with PTC in our institution were retrospectively collected. Patients with tumors less than 10 mm in diameter were classified as PTMC cohort and the rest as PTC cohort. Clinicopathological features between PTMC and PTC were compared on the basis of SEER cohort and validated with institutional data. Survival analysis was conducted to explore the effect of surgery on the prognosis of patients. To minimize potential confounders and selection bias, we performed propensity score matching (PSM) analysis to match more comparable cohorts.

**Results:**

Compared with PTC, PTMC exhibited the following characteristics: more common in women and whites, older age at diagnosis, lower proportion of follicular variants, intraglandular dissemination, extraglandular and capsular invasion, higher proportion of multifocality, fewer lymph node and distant metastases, and higher cancer-specific survival (CSS) and overall survival (OS) (all *p*-value < 0.05). Regarding treatment, patients with PTMC received a lower proportion of radiotherapy, chemotherapy, and total thyroidectomy but a higher proportion of lobectomy and/or isthmectomy. There was no significant difference in CSS for patients with PTMC at stage T1N0M0 with or without surgery (*P* = 0.36).

**Conclusion:**

Generally, PTMC showed higher biological indolence than PTC, which meant a higher survival rate for patients in both OS and CSS. For patients with PTMC at staged T1N0M0, active surveillance (AS) may be a potentially feasible management strategy. However, the maintenance of good medical compliance and the management of psychological burden cannot be ignored for patients included in AS.

## Background

Thyroid cancer (THCA) is the most common endocrine malignancy, increasing at incidence rate of at least 4.5% per year (1). It has been estimated that THCA will become the top four most common malignancy by 2030 (2). Papillary thyroid carcinoma (PTC) is the most common subtype of THCA, comprising 80% of all cases (3). Papillary thyroid microcarcinoma (PTMC) is PTC with a maximum tumor diameter of less than 10 mm (4), which is considered to significantly contribute to the increasing incidence of THCA (5). The current management standard for PTC is immediate surgical treatment, including total thyroidectomy with bilateral central lymph node dissection (CLND), unilateral thyroid lobectomy with CLND, or total thyroidectomy with bilateral CLND and lateral cervical LN dissection of the affected side. However, despite the incidence of PTC increasing, its mortality rate has remained relatively stable (6, 7). It was reported that, due to the indolence of PTC, most PTMC would either not progress or progress so slowly that it never leads to clinically meaningful disease over the patient’s lifetime (8). In addition, the prevalence of occult PTC at autopsy in the general population was high, at 35.6% (9). Therefore, considering the very low disease-specific mortality and recurrence rates of PTMC and risk of surgical complications, the traditional management approach of immediate thyroid surgery for PTMC is being reconsidered (10). Currently, several studies have focused on the clinicopathological differences between PTC and PTMC and whether surgery is the preferred treatment by all patients with PTC. Although active surveillance (AS) was included in clinical guidelines in Japan in 2010 (11) and the United States in 2015 (12) as an alternative management strategy for low-risk PTMC, this strategy is still questioned by many thyroid doctors and patients due to the limited data available (13).

Propensity score matching (PSM) is a statistical method to ensure an even distribution of confounders and biases between treatment and control cohorts, improving comparability between cohorts (14). It has emerged as the preferred method of matching exposed and nonexposed patients in observational cohort studies, yielding estimates of effects similar to the results of randomized control trials (15). Therefore, using PSM, this study aimed to compare clinicopathological features between PTMC and PTC and explore whether surgery can confer significant survival benefits in all patients with PTC or PTMC.

## Patients and methods

### Data source and patient selection

Data of patients with PTC were extracted from 18 population-based cancer registries of the SEER database (https://seer.cancer.gov/) from 2000 through 2018 using the SEER*Stat program (version 8.3.9). The SEER database was a cancer incidence registry including about 30% of the US population. The extraction criteria were as follows: “Primary Site = C73.9-Thyroid gland” and “Behavior code ICD-0-3 = Malignant”. The exclusion criteria were as follows: (1) nonpathological diagnosis; (2) non-papillary carcinoma as the histologic type; (3) unknown survival time; (4) unknown tumor size; (5) unknown surgery approach; and (6) unknown if surgery underwent. The variables extracted from eligible cases included the following: patient ID, age at diagnosis, sex, race/ethnicity, year of diagnosis, follicular variant, multifocality, laterality, tumor size, T stage, N stage, M stage, surgery of primary site, radiotherapy recode, chemotherapy recode, distant metastases record, number of lesions, follow-up months, number of metastatic lymph node (LN), distant metastasis record, SEER cause-specific death classification, and vital status recode (study cutoff used). In addition, data of patients with PTC diagnosed and treated in our institution from 2009 to 2020 were collected. Inclusion criteria were as follows: (1) pathological diagnosis was PTC; (2) clinicopathological information was complete and available; (3) surgery was underwent; and (4) informed consent was signed. The parameters collected included the following: patient ID, sex, age, year of diagnosis, surgery approach, laterality, follicular subtype, multifocality, intraglandular dissemination, extraglandular invasion, capsular invasion, T stage, N stage, and M stage. The demographic and clinicopathological data of all eligible cases were retrospectively analyzed.

### Cohort definition and variable recode

All eligible patients with PTC were divided into PTMC and PTC cohort according to whether tumor diameter ≤10 mm and then matched with propensity scores to obtain more comparable cohorts. In addition, patients with PTC or PTMC were divided into two cohorts according to whether they underwent surgery or not to explore the effect of surgery on prognosis of patients. The variables analyzed included all factors, age (≤55 or >55), sex (female or male), race (black, white, or other), follicular variant (yes or no), multifocality (yes or no), T stage, N stage, M stage, radiotherapy (yes or no), chemotherapy (yes or no), and surgery approach. The cutoff point for continuous variables such as age was generated by the “surv_cutpoint” function of the “Survminer” package in R. The primary outcomes investigated were cancer-specific survival (CSS) and overall survival (OS). CSS was classified on the basis of available death certificate information using SEER-defined variables. OS was defined as the time from diagnosis until death or last follow-up.

### Study design

First, after initial screening, patients with PTC and PTMC were classified by year of diagnosis to roughly estimate incidence trends of PTC and PTMC over time. Second, statistical differences in clinicopathological parameters of the PTC and PTMC cohorts from the SEER database were compared before and after PSM and validated with our institution’s cohort. Third, survival analysis was performed to compare the prognosis (both CSS and OS) between patients with PTC and those with PTMC, and between patients who underwent and those who did not perform surgery. Finally, the cohort of to whom surgery was recommended but not underwent was further selected and then matched with the cohort that underwent surgery using PSM. In addition, and survival analysis was applied to evaluate the difference in prognosis. This work has been reported following the STROCSS criteria (16).

### Propensity score matching analysis

PSM analysis was used to match patients with PTC and those with PTMC. This method was also used to match patients who underwent surgery and those who were recommended but did not, to balance the potential baseline confounding factor (17). The “Matchit” package in R studio (http://www.r-project.org) was used to match the propensity score between cohorts, and the matching approach was set as the nearest neighbor algorithm with a matching ratio of 1:1 and a caliper value of 0.01 (18). The variables balanced using PSM included age, sex, race, year of diagnosis, laterality, multifocality, and follicular variant. The “Cobalt” package of R studio was applied to estimate kernel density and analyze standardized difference of means and to evaluate the covariate balance before and after matching (19). An absolute value of the mean difference less than 0.05 was considered as qualified matching.

### Statistical analysis

Continuous variables with normal distribution were expressed as mean and standard deviation (SD) and as median and interquartile range (IQR) for nonnormally distributed variables. The Student’s t-test (normally distributed) or Mann-Whitney U-test (nonnormally distributed) was used to compare continuous variables. Categorical variables were presented as frequencies and percentages (%) and analyzed using Fisher’s exact test or Pearson χ2 test. A two-sided *P*-value <0.05 was considered statistically significant. R studio version 4.0.3 software (http://www.r-project.org) was used to perform all statistical analyses and visualization.

### Ethics statement

This study was exempt from the approval processes of the Institutional Review Boards because no personal information about patients was sought and their identities will not be revealed in any publication. Informed consent was obtained from all patients for additional personal data.

## Results

### Demographic and clinicopathological characteristics of patients

**Figure 1:**
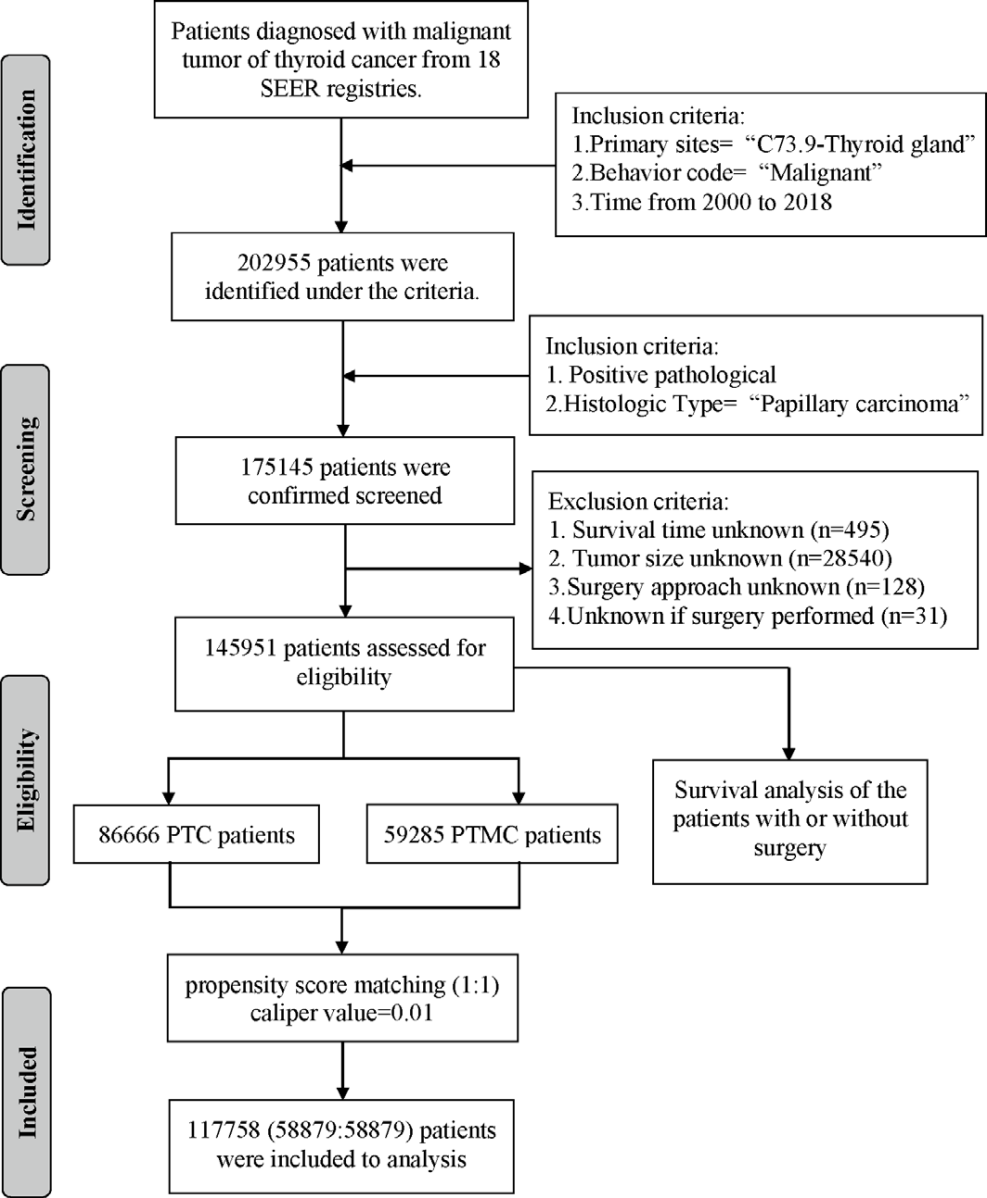
Flow diagram presenting the screening process in the SEER database.

**Figure 2:**
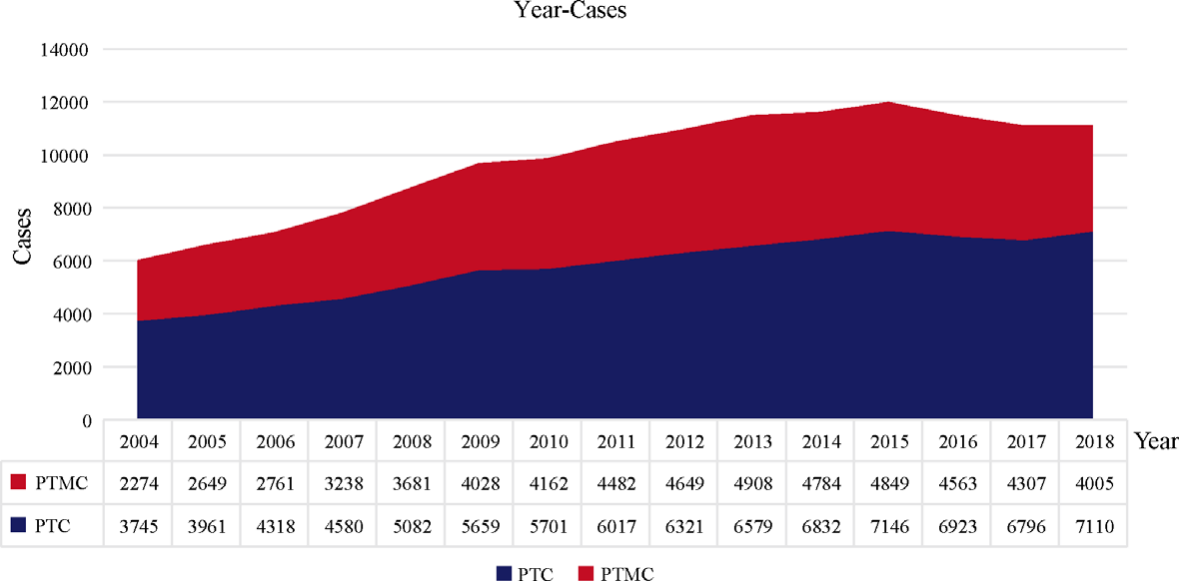
The number of PTC and PTMC cases diagnosed annually recorded in the SEER database consisting of 18 population-based cancer registries. PTC, papillary thyroid carcinoma; PTMC, papillary thyroid microcarcinoma.

A total of 145,951 patients registered in the SEER database from 2004 to 2018 and 8,751 patients treated at our center from 2009 to 2020 were included in this study. The detailed flow diagram showing the patient inclusion and exclusion criteria process in the SEER database is shown in **Figure 1**. The number of diagnosed cases per year is shown in **Figure 2**, indicating that the incidence of both PTC and PTMC was increased year by year. The mean age at diagnosis was 48.3 years (SD = 16.0) and 51.2 years (SD = 14.1) for PTC and PTMC, respectively. The median follow-up time was 66 months for PTC cohort (IQR: 29–111) and 70 months (IQR: 34–112) for PTC cohort. Compared with PTC, PTMC exhibited the following characteristics: more common in women (81.1% vs. 74.1%) and whites (82.2% vs. 80.1%), lower proportion of follicular variants (27.9% vs. 31.0%), higher proportion of multifocality (19.9% vs. 16.8%), and higher CSS (99.7% vs. 98.3%) and OS (94.3% vs. 92.7%) (all *p*-value <0.001).

Validation data from our institution also confirmed that, compared with PTC, PTMC was more common in women (80.6% vs. 74.5%) and had older age at diagnosis (44.79 vs. 42.87), higher proportion of bilateral lesions (28.1% vs. 22.3%), lower proportion of follicular subtype (0.7% vs. 1.7%), and higher proportion of lobectomy and/or isthmectomy (21.3% vs. 14.7%) but lower proportion of total thyroidectomy (78% vs. 83%) (all the *p*-value<0.001). Detailed data are presented in **Table 2**.

### PSM adjustment of patient characteristics

Patients with PTMC and PTC were 1:1 propensity matched with a caliper value of 0.01 to yield 58,879 matched pairs of patients. The clinicopathological comparison between the two cohorts is presented in **Table 1** and **Supplementary Table 1**. After minimizing potential bias using PSM analysis, PTMC still showed consistently lower T, N, and M stages and fewer LN (11.9% vs. 30.8%) and distant metastases (0.3% vs. 1.5%) compared with PTC. In addition, the difference between PTMC and PTC in OS (94.3% vs. 91.9%) and CSS (99.7% vs. 98.1%) was more significant (all *p*-value <0.001). Meanwhile, as shown in **Supplementary Table 1**, compared with PTMC, PTC had more lung (0.7% vs. 0.1%), bone (0.3% vs. 0.1%), and distant LN (0.1% vs. 0) metastases. Together, these results meant that PTMC had a higher biological indolence than PTC. Regarding treatment, patients with PTMC had a lower proportion of radiotherapy (24.3% vs. 59.5%), chemotherapy (0.1% vs. 0.6%), and total thyroidectomy (72.2% vs. 87.3%) but a higher proportion of lobectomy and/or isthmectomy (21.5% vs. 7%) compared with patients with PTC (all *p-*value <0.001).

**Table 1:** Clinicopathological characteristics and statistical results of patients with PTC or PTMC recorded in SEER database before and after propensity score matching.

Characteristic	Level	Pre–Propensity Score Matching				Post–Propensity Score Matching			
		Overall	PTC	PTMC	P	Overall	PTC	PTMC	P
N		145,951	86,666	59,285		117,758	58,879	58,879	
Age, mean (SD)		49.4 (15.34)	48.3 (16.0)	51.2 (14.1)	<0.001	51.3 (14.5)	51.4 (14.9)	51.1 (14.1)	<0.001
Age group, n (%)	≤55 years	94,141 (64.5)	57,925 (66.8)	36,216 (61.1)	<0.001	71,811 (61.0)	35,670 (60.6)	36,141 (61.4)	0.005
	>55 years	51,810 (35.5)	28,741 (33.2)	23,069 (38.9)		45,947 (39.0)	23,209 (39.4)	22,738 (38.6)	
Sex, n (%)	Female	112,296 (76.9)	64,204 (74.1)	48,092 (81.1)	<0.001	95,215 (80.9)	47,516 (80.7)	47,699 (81.0)	0.178
	Male	33,655 (23.1)	22,462 (25.9)	11,193 (18.9)		22,543 (19.1)	11,363 (19.3)	11,180 (19.0)	
Race, n (%)	Black	9,224 (6.3)	5,207 (6.0)	4,017 (6.8)	<0.001	7,566 (6.4)	3,579 (6.1)	3,987 (6.8)	<0.001
	Other	18,595 (12.7)	12,077 (13.9)	6,518 (11.0)		13,134 (11.2)	6,621 (11.2)	6,513 (11.1)	
	White	118,132 (80.9)	69,382 (80.1)	48,750 (82.2)		97,058 (82.4)	48,679 (82.7)	48,379 (82.2)	
Follicular variant, n (%)	No	102,505 (70.2)	59,762 (69.0)	42,743 (72.1)	<0.001	84,431 (71.7)	42,031 (71.4)	42,400 (72.0)	0.017
	Yes	43,446 (29.8)	26,904 (31.0)	16,542 (27.9)		33,327 (28.3)	16,848 (28.6)	16,479 (28.0)	
Multifocality, n (%)	No	119,604 (81.9)	72,130 (83.2)	47,474 (80.1)	<0.001	94,681 (80.4)	47,566 (80.8)	47,115 (80.0)	0.001
	Yes	26,347 (18.1)	14,536 (16.8)	11,811 (19.9)		23,077 (19.6)	11,313 (19.2)	11,764 (20.0)	
Laterality (%)	A	144,908 (99.3)	85,948 (99.2)	58,960 (99.5)	<0.001	117,184 (99.5)	58,630 (99.6)	58,554 (99.4)	0.002
	B	1,043 (0.7)	718 (0.8)	325 (0.5)		574 (0.5)	249 (0.4)	325 (0.6)	
T stage, n (%)	T1	92,877 (63.6)	36,504 (42.1)	56,373 (95.1)	<0.001	81,569 (69.3)	25,582 (43.4)	55,987 (95.1)	<0.001
	T2	25,082 (17.2)	25,082 (28.9)	0 (0.0)		16,321 (13.9)	16,321 (27.7)	0 (0.0)	
	T3&T4	27,805 (19.1)	24,893 (28.7)	2,912 (4.9)		19,747 (16.8)	16,855 (28.6)	2,892 (4.9)	
	Tx	187 (0.1)	187 (0.2)	0 (0.0)		121 (0.1)	121 (0.2)	0 (0.0)	
N stage, n (%)	N0	99,385 (68.1)	52,560 (60.6)	46,825 (79.0)	<0.001	83,103 (70.6)	36,631 (62.2)	46,472 (78.9)	<0.001
	N1	34,964 (24.0)	27,923 (32.2)	7,041 (11.9)		25,145 (21.4)	18,133 (30.8)	7,012 (11.9)	
	Nx	11,602 (7.9)	6,183 (7.1)	5,419 (9.1)		9,510 (8.1)	4,115 (7.0)	5,395 (9.2)	
M stage, n (%)	M0	143,072 (98.0)	84,367 (97.3)	58,705 (99.0)	<0.001	115,676 (98.2)	57,374 (97.4)	58,302 (99.0)	<0.001
	M1	1,513 (1.0)	1,340 (1.5)	173 (0.3)		1,057 (0.9)	885 (1.5)	172 (0.3)	
	Mx	1,366 (0.9)	959 (1.1)	407 (0.7)		1,025 (0.9)	620 (1.1)	405 (0.7)	
Radiotherapy, n (%)	Yes	65,823 (45.1)	51,436 (59.3)	14,387 (24.3)	<0.001	49,365 (41.9)	35,055 (59.5)	14,310 (24.3)	<0.001
	No	80,128 (54.9)	35,230 (40.7)	44,898 (75.7)		68,393 (58.1)	23,824 (40.5)	44,569 (75.7)	
Surgery approach, n (%)	No surgery or surgery, NOS	2,705 (1.9)	2,125 (2.5)	580 (1.0)	<0.001	2,092 (1.8)	1,523 (2.6)	569 (1.0)	<0.001
	Local tumor resection	1,033 (0.7)	396 (0.5)	637 (1.1)		880 (0.7)	252 (0.4)	628 (1.1)	
	Lobectomy and/or isthmectomy	19,181 (13.1)	6,450 (7.4)	12,731 (21.5)		16,756 (14.2)	4,103 (7.0)	12,653 (21.5)	
	Near total thyroidectomy	4,912 (3.4)	2,381 (2.7)	2,531 (4.3)		4,091 (3.5)	1,576 (2.7)	2,515 (4.3)	
	Total thyroidectomy	118,120 (80.9)	75,314 (86.9)	42,806 (72.2)		93,939 (79.8)	51,425 (87.3)	42,514 (72.2)	
Chemotherapy, n(%)	Yes	513 (0.4)	430 (0.5)	83 (0.1)	<0.001	408 (0.3)	325 (0.6)	83 (0.1)	<0.001
	No	145,438 (99.6)	86,236 (99.5)	59,202 (99.9)		117,350 (99.7)	58,554 (99.4)	58,796 (99.9)	
Cancer-specific death, n(%)	Yes	1,643 (1.1)	1,490 (1.7)	153 (0.3)	<0.001	1,299 (1.1)	1,146 (1.9)	153 (0.3)	<0.001
	No	144,308 (98.9)	85,176 (98.3)	59,132 (99.7)		116,459 (98.9)	57,733 (98.1)	58,726 (99.7)	
Status, n(%)	Dead	9,711 (6.7)	6,312 (7.3)	3,399 (5.7)	<0.001	8,134 (6.9)	4,764 (8.1)	3,370 (5.7)	<0.001
	Alive	136,240 (93.3)	80,354 (92.7)	55,886 (94.3)		109,624 (93.1)	54,115 (91.9)	55,509 (94.3)	

PTC, papillary thyroid carcinoma; PTMC, papillary thyroid microcarcinoma; N/n, number; SD, standard deviation; NOS, not otherwise specified.

Validation data from our center also verified that, after PSM, PTC showed stably higher T staging and N staging and higher proportion of intraglandular dissemination (4.7% vs. 2.8%), extraglandular invasion (5.8% vs. 1.1%), and capsular invasion (55.9% vs. 42.1%) (all *p*-value <0.001), which supported the results of SEER data analysis. Detailed data are presented in **Table 2**. In addition, the comparison of clinicopathological features between the cohorts (patients who underwent surgery and those who were recommended but did not undergo surgery) is presented in **Supplementary Table 2**.

**Table 2:** Clinicopathological characteristics and statistical results of patients with PTC or PTMC recorded in our institution before and after propensity score matching.

Characteristic	Level	Pre–Propensity Score Matching				Post–Propensity Score Matching			
		Overall	PTC	PTMC	P	Overall	PTC	PTMC	P
N		8,751	4,456	4,295		6,998	3,499	3,499	
Age, mean (SD)		43.81 (11.31)	42.87 (11.93)	44.79 (10.54)	<0.001	43.92 (10.96)	43.98 (11.49)	43.87 (10.39)	0.689
Age group, n (%)	≤55 year	7,474 (85.4)	3,816 (85.6)	3,658 (85.2)	0.555	6,000 (85.7)	2,954 (84.4)	3,046 (87.1)	0.002
	>55 year	1,277 (14.6)	640 (14.4)	637 (14.8)		998 (14.3)	545 (15.6)	453 (12.9)	
Sex, n (%)	Male	1,968 (22.5)	1,135 (25.5)	833 (19.4)	<0.001	1,530 (21.9)	765 (21.9)	765 (21.9)	1
	Female	6,783 (77.5)	3,321 (74.5)	3,462 (80.6)		5,468 (78.1)	2,734 (78.1)	2,734 (78.1)	
Laterality, n (%)	Unilateral	6,550 (74.8)	3,464 (77.7)	3,086 (71.9)	<0.001	5,186 (74.1)	2,636 (75.3)	2,550 (72.9)	0.02
	Bilateral	2,201 (25.2)	992 (22.3)	1,209 (28.1)		1,812 (25.9)	863 (24.7)	949 (27.1)	
Surgery approach, n (%)	Surgery, NOS	69 (0.8)	64 (1.4)	5 (0.1)	<0.001	55 (0.8)	52 (1.5)	3 (0.1)	<0.001
	Local tumor resection	8 (0.1)	8 (0.2)	0 (0.0)		6 (0.1)	6 (0.2)	0 (0.0)	
	Lobectomy and/or isthmectomy	1,569 (17.9)	654 (14.7)	915 (21.3)		1,260 (18.0)	490 (14.0)	770 (22.0)	
	Near total thyroidectomy	55 (0.6)	30 (0.7)	25 (0.6)		41 (0.6)	22 (0.6)	19 (0.5)	
	Total thyroidectomy	7,050 (80.6)	3,700 (83.0)	3,350 (78.0)		5,636 (80.5)	2,929 (83.7)	2,707 (77.4)	
T stage, n (%)	T1	7,231 (82.6)	3,352 (75.2)	3,879 (90.3)	<0.001	5,773 (82.5)	2,611 (74.6)	3,162 (90.4)	<0.001
	T2	385 (4.4)	382 (8.6)	3 (0.1)		290 (4.1)	288 (8.2)	2 (0.1)	
	T3	579 (6.6)	383 (8.6)	196 (4.6)		457 (6.5)	319 (9.1)	138 (3.9)	
	T4	556 (6.4)	339 (7.6)	217 (5.1)		478 (6.8)	281 (8.0)	197 (5.6)	
N stage, n (%)	N0	4,446 (50.8)	1,727 (38.8)	2,719 (63.3)	<0.001	3,563 (50.9)	1,397 (39.9)	2,166 (61.9)	<0.001
	N1	4,305 (49.2)	2,729 (61.2)	1,576 (36.7)		3,435 (49.1)	2,102 (60.1)	1,333 (38.1)	
M stage, n (%)	M0	8,722 (99.7)	4,442 (99.7)	4,280 (99.7)	0.921	6,980 (99.7)	3,491 (99.8)	3,489 (99.7)	0.813
	M1	29 (0.3)	14 (0.3)	15 (0.3)		18 (0.3)	8 (0.2)	10 (0.3)	
Follicular subtype, n (%)	No	8,645 (98.8)	4,381 (98.3)	4,264 (99.3)	<0.001	6,953 (99.4)	3,478 (99.4)	3,475 (99.3)	0.765
	Yes	106 (1.2)	75 (1.7)	31 (0.7)		45 (0.6)	21 (0.6)	24 (0.7)	
Multifocality, n (%)	No	5,432 (62.1)	2,771 (62.2)	2,661 (62.0)	0.842	4,317 (61.7)	2,138 (61.1)	2,179 (62.3)	0.325
	Yes	3,319 (37.9)	1,685 (37.8)	1,634 (38.0)		2,681 (38.3)	1,361 (38.9)	1,320 (37.7)	
Intraglandular dissemination, n (%)	No	2,969 (33.9)	1,826 (41.0)	1,143 (26.6)	<0.001	2,332 (33.3)	1,343 (38.4)	989 (28.3)	<0.001
	Unknown	5,434 (62.1)	2,402 (53.9)	3,032 (70.6)		4,402 (62.9)	1,990 (56.9)	2,412 (68.9)	
	Yes	348 (4.0)	228 (5.1)	120 (2.8)		264 (3.8)	166 (4.7)	98 (2.8)	
Extratglandular invasion, n (%)	No	7,913 (90.4)	3,801 (85.3)	4,112 (95.7)	<0.001	6,312 (90.2)	2,972 (84.9)	3,340 (95.5)	<0.001
	Unknown	547 (6.3)	408 (9.2)	139 (3.2)		445 (6.4)	325 (9.3)	120 (3.4)	
	Yes	291 (3.3)	247 (5.5)	44 (1.0)		241 (3.4)	202 (5.8)	39 (1.1)	
Capsular invasion, n (%)	No	3,900 (44.6)	1,557 (34.9)	2,343 (54.6)	<0.001	3,123 (44.6)	1,217 (34.8)	1,906 (54.5)	<0.001
	Unknown	547 (6.3)	408 (9.2)	139 (3.2)		445 (6.4)	325 (9.3)	120 (3.4)	
	Yes	4,304 (49.2)	2,491 (55.9)	1,813 (42.2)		3,430 (49.0)	1,957 (55.9)	1,473 (42.1)	

PTC, papillary thyroid carcinoma; PTMC, papillary thyroid microcarcinoma; N/n, number; SD, standard deviation.

### Assessment of covariate balance in matched groups

**Figure 3:**
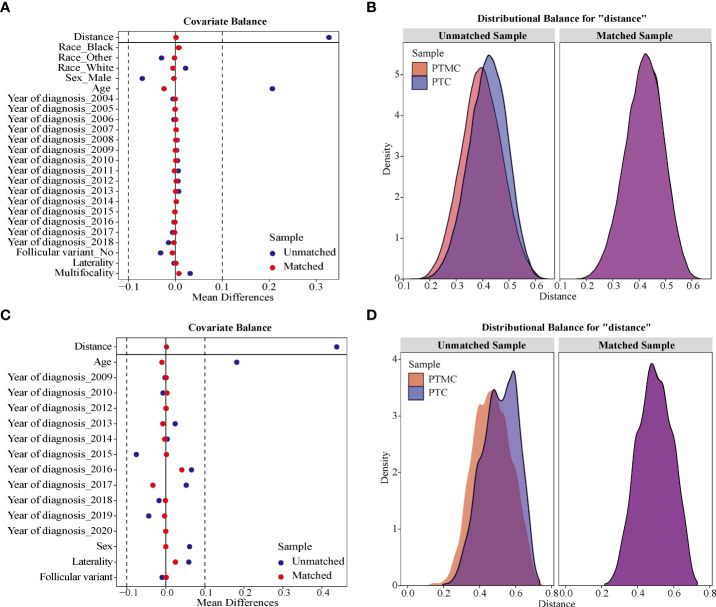
The assessment of sample distribution and covariate balance before and after propensity score matching. **(A, B)** For the data from SEER database and **(C, D)** for the data from our institution. **(A, C)** Mean standardized difference analysis showed that all matched covariates were less than 10%, indicating good matching effect. **(B, D)** Kernel density estimation indicated that, compared with the unmatched sample, the matched sample showed almost complete overlap, suggesting better comparability between the two cohorts.

Assessment of the covariate balance in the matched cohorts was an important step to diagnose the quality of matched samples (20). In present study, the balance was defined as similarity in the empirical distributions of the full set of covariates between matched PTMC and PTC cohorts. As shown in **Figures 3A, C** analysis of standardized mean difference suggested that almost all covariates were less than 5%, far less than the 10% reported in the literature (21, 22), suggesting a good match. In addition, Kernel density estimation in **Figures 3B, D** indicated that, compared with the unmatched sample, the matched sample showed almost complete overlap, suggesting better comparability between the two cohorts. Therefore, our results jointly confirmed that the confounders and bias between the cohorts were well balanced, and the comparison was reliable.

### Survival analysis outcomes in patients with PTC vs. those with PTMC

**Figure 4:**
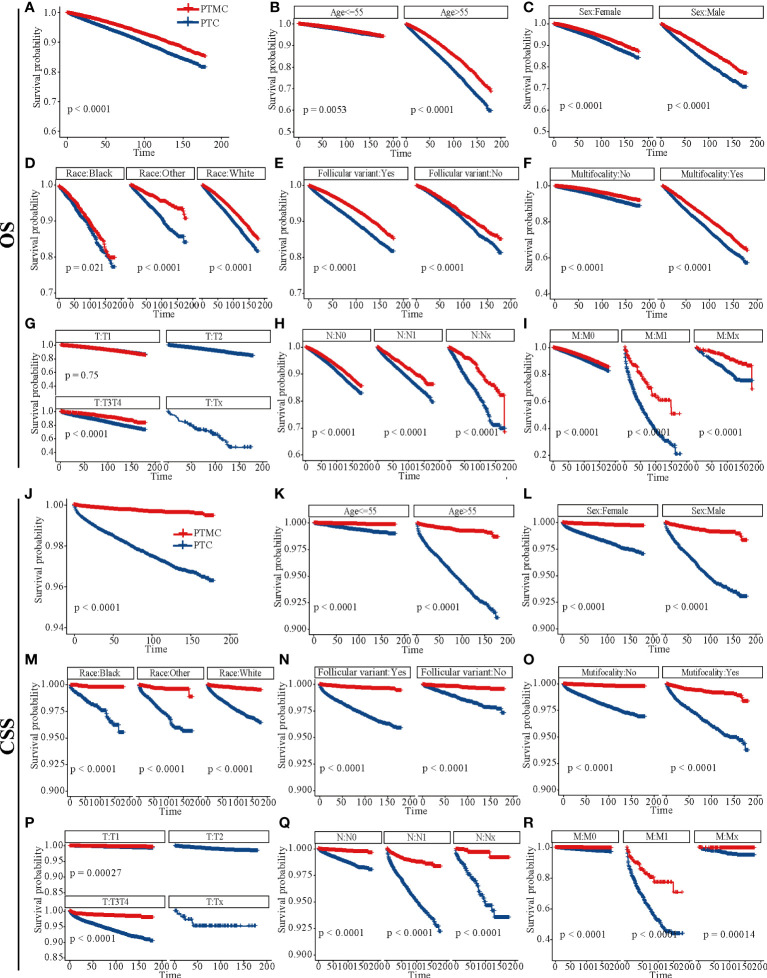
Survival analysis of OS and CSS for patients with PTC and PTMC at different stages or with different characteristics. **(A–I)** OS analysis and **(J–R)** CSS analysis. **(A, J)** All factors; **(B, K)** age; **(C, L)** sex; **(D, M)** race; **(E, N)** follicular variant; **(F, O)** multifocality; **(G, P)** T stage; **(H, Q)** N stage; **(I, R)** M stage. OS, overall survival; CSS, cancer-specific survival; PTC, papillary thyroid carcinoma; PTMC, papillary thyroid microcarcinoma.

The survival analysis in **Figure 4** suggested that the 5-year OS and 10-year OS of PTC were about 94% and 88%, respectively, and that of PTMC were about 96% and 91%, respectively. In addition, the 5-year CSS and 10-year CSS of PTC were about 98% and 97%, respectively, whereas the 5-year and 10-year CSS of PTMC were above 99%. Detailed data are presented in **Table 3**. In addition, PTMC showed a better prognosis than PTC for both OS and CSS under similar clinicopathological parameters such as age, sex, race, follicular variant, multifocality, and TNM staging (all *p*-value < 0.01).

**Table 3:** An overall estimation of prognosis for different types of patients based on survival analysis.

Categories	OS		CSS	
	5-year	10-year	5-year	10-year
PTC	94%	88%	98%	97%
PTMC	96%	91%	>99%	>99%
Patients with PTC with surgery	95%	90%	99%	98%
Patients with PTC without surgery	62%	50%	86%	82%
Patients with PTMC with surgery	97%	92%	>99%	>99%
Patients with PTMC without surgery	76%	67%	98%	98%

PTC, papillary thyroid carcinoma; PTMC, papillary thyroid microcarcinoma; OS, overall survival; CSS, cancer-specific survival.

### Survival analysis outcomes in patients with and without surgery

**Figure 5:**
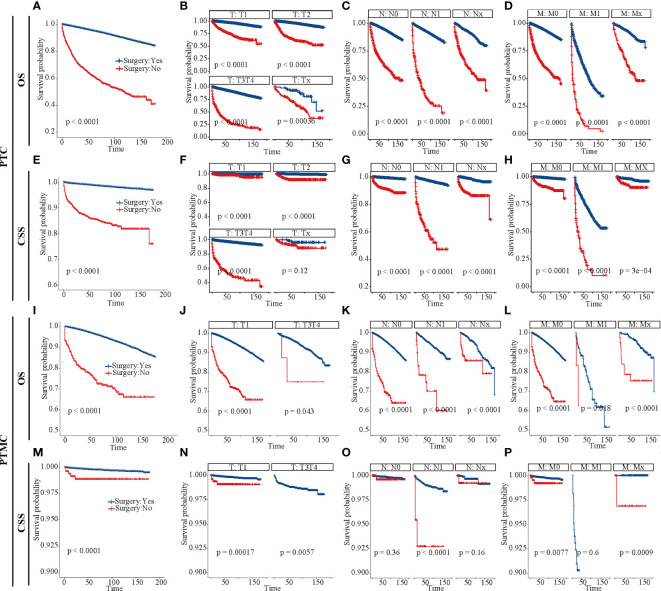
Survival analysis of patients with PTC and PTMC with or without surgery at different stages. **(A–D)** OS for patients with PTC; **(E–H)** CSS for patients with PTC; **(I–L)** OS for patients with PTMC; **(M–P)**: CSS for patients with PTMC. **(A, E, I, M)** All factors; **(B, F, J, N)** T stage; **(C, G, K, O)** N stage; **(D, H, L, P)** M stage. OS, overall survival; CSS, cancer-specific survival; PTC, papillary thyroid carcinoma; PTMC, papillary thyroid microcarcinoma.

Survival analysis in **Figure 5** showed that the 5-year OS, 5-year CSS, 10-year OS, and 10-year CSS were about 95%, 99%, 90%, and 98%, respectively, in patients with PTC who underwent surgery, compared with about 62%, 86%, 50% and 82%, respectively, in patients with PTC without surgery. Similarly, the 5-year OS and 10-year OS of PMTC patients who underwent surgery were about 97% and 92%, respectively, whereas both the 5-year and 10-year CSS were above 99%. However, the 5-year OS and 10-year OS of patients with PTMC who did not undergo surgery were about 76% and 67%, respectively, whereas both the 5-year and 10-year CSS were about 98%. Detailed results are presented in **Table 3**.

**Figure 6:**
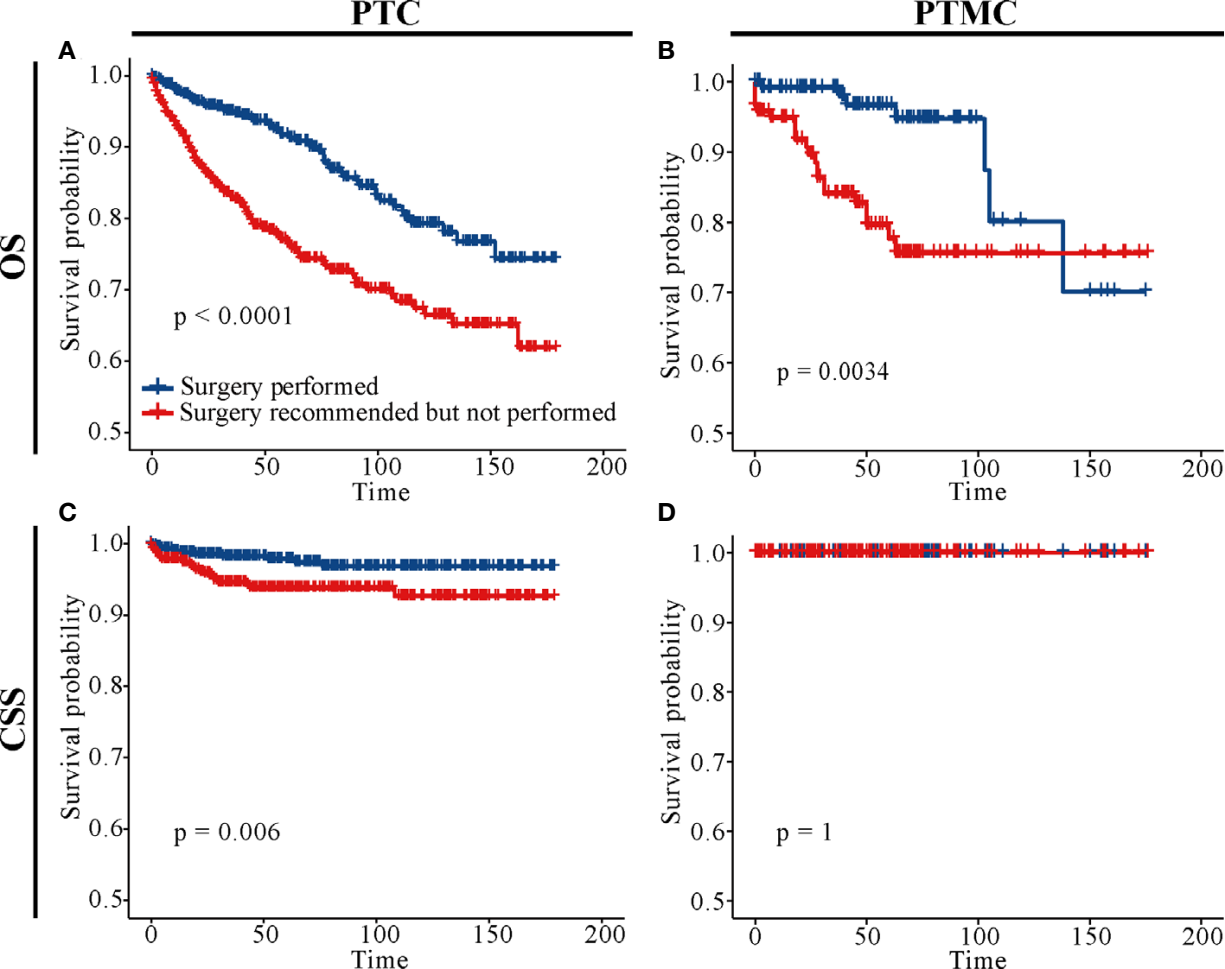
Survival analysis of patients with PTC and PTMC who underwent surgery and those who were recommended but did not undergo surgery. **(A)** OS for PTC patients; **(B)** OS for patients with PTMC; **(C)** CSS for PTC patients; **(D)** CSS for patients with PTMC; OS, overall survival; CSS, cancer-specific survival; PTC, papillary thyroid carcinoma; PTMC, papillary thyroid microcarcinoma.

In addition, analysis of patients at different TNM stages suggested that surgery improved CSS and OS in almost all patients (all *p*-value < 0.05), except patients with PTMC at stage T1N0M0. **Figure 5O** indicated that there was no significant difference in CSS for patients with PTMC at stage T1N0M0 with and without surgery (*p* = 0.36), although OS was statistically significant for this group of patients (*p* < 0.0001). As shown in **Figure 6D**, further survival analysis of patients underwent surgery and those who were recommended but did not undergo surgery also showed no statistical difference in CSS for patients with PTMC (*p* = 1), and no patients died due to PTMC in both cohorts. Therefore, for patients with PTMC at stage T1N0M0, AS with good medical compliance may be a potentially feasible management strategy. However, for PTC patients, the differences were significant for both OS and CSS (**Figures 6A, C**).

## Discussion

In recent years, with the increasing incidence of THCA but stable mortality (7), overtreatment of PTC has become a major concern. Consequently, an increasing number of studies are exploring the feasibility of AS in PTC treatment. In this study, using PSM, we elucidated clinicopathological differences between PTC and PTMC based on data of 145,951 patients with PTC from the SEER database and validated them with data of 8,751 patients with PTC in our institution. It was concluded that PTMC generally presented higher biological indolence than PTC, which meant a higher OS and CSS rate for patients. In addition, survival analysis suggests no significant difference in CSS between patients with PTMC at stage T1N0M0 with and without surgery (*p* = 0.36). This meant that surgery could not confer significant survival benefits for these patients, and AS may be a potentially more viable alternative management strategy than surgery. Taken together, the results of this population-based PSM analysis confirm differences in the biological behavior and prognosis of PTC and PTMC and provide evidence for AS in patients with PTMC at stage T1N0M0.

Although both PTMC and PTC are considered indolent malignancy (23), they receive uniform immediate surgical treatment with no differences in management strategies. Hence, identifying clinicopathological differences between the two could improve management of PTC. This study suggested that PTMC was diagnosed at an older age and had a lower proportion of follicular variants, intraglandular dissemination, extraglandular invasion, and capsular invasion than PTC (**Tables 1**, **2**). Previous studies have identified younger age (≤45 years) at diagnosis, extraglandular invasion and capsular invasion as risk factors for PTC recurrence (24) and LN metastases (19, 25, 26). Ito et al. concluded that PTMC may be more progressive in young patients than in older patients (27). In addition, a previous report confirmed that PTC displays a distinct gene expression pattern from PTC (28). Thus, these clinicopathological features may partly elucidate the higher biological indolence of PTMC, OS, and CSS compared with PTC. In addition, as the tumor was at an earlier stage and the risk was lower, patients with PTMC received a lower proportion of radiotherapy, chemotherapy, and total thyroidectomy but a higher proportion of lobectomy and/or isthmectomy than PTC patients. In addition, both data from the SEER database and our institution suggested that PTMC exhibited higher proportion of multifocality compared with PTC, consistent with the high multifocality of PTMC reported in the literature (29). Research suggested that even patients with multiple PTMC were good candidates for AS (30).

Although AS for low-risk PTMC was introduced into the management guidelines of THCA in Japan and the United States in 2010 and 2015, respectively, long-term follow-up data for patients included in AS are still limited. As a result, some clinicians or patients are reluctant to perform it. This study retrospectively analyzed long-term follow-up results of patients with or without surgery, providing an evidence for clinical management of PTC and PTMC. As shown in **Figure 5O**, CSS was not significant different between patients with PTMC at stage T1N0M0 with and without surgery. This result was consistent with the Consensus Statements from the Japan Association of Endocrine Surgery Task Force on Management for Papillary Thyroid Microcarcinoma (31). As shown in **Figure 6D**, further survival analysis showed no statistical difference in CSS between patients who underwent surgery and those who were recommended but did not undergo surgery for patients with PTMC (*p* = 1). Further, no patients died of PTMC in both cohorts. Similarly, in a prospective study of 230 patients with asymptomatic PTMC, 90% of PTMC remained stable during the 17-year observation period, and no patient developed distant metastasis or died due to PTMC (32). Moreover, a study of quality of life of patients with low-risk PTMC reported that patients in the immediate surgery group had more complaints and were more anxious and depressed than those in the AS group (33). In a cohort of patients with PTMC observed for more than 50 years, only three patients (0.3%) died of PTMC, compared with 29% of patients dying of other causes (34). Consistently, patients with PTMC at stage T1N0M0 who did not undergo surgery had significantly lower OS than those who underwent it (**Figures 5K**, **6B**). This may be partly due to excessive psychological stress and poor medical compliance. Therefore, it was necessary to eliminate the psychological burden of patients with PTMC under AS, urge them to carry out standardized AS and maintain good medical compliance, and minimize the effect of comorbidities. Appropriate medical education of the patients may reduce cancer and surveillance-related stress in patients, facilitate patient compliance with AS protocols, and improve follow-up (10). In conclusion, for patients with PTMC at stage T1N0M0, AS may be a potentially feasible management strategy.

AS is defined in literature as the life-long application of meticulous diagnostic modalities to monitor changes in the status of a disease without immediate therapeutic measures until the progression of the disease is evident, through regular testing and assessment of cancer progression (35). It has been successfully applied to the prostate cancer, which is also considered low risk (36). Ito et al. proposed the following strategy of AS for low-risk PTMC: Patients enrolled in AS were required to revisit hospital after 6 months, and if no signs of progression such as enlargement (increase by more than 3mm) or LN metastases were detected, then their next visit was scheduled 1 year later (4). Otherwise AS would be terminated, and immediate surgery would be performed. In addition, if the patient felt overwhelmed with the psychological burden of not undergoing treatment or changed preferences, then he or she should be switched to surgery therapy. By comparison, several suggestions for AS have been proposed in literature (10, 31): (1) accurately identify and exclude aggressive tumors at the beginning and during AS; (2) develop a standardized thyroid ultrasound examination and recording system; (3) provide appropriate education of patients and clinicians to fully understand follow-up protocols; (4) evaluate factors affecting patient compliance with follow-up; and (5) provide necessary psychological support to ensure a good quality of life.

This study has limitations that must be acknowledged. First, as a retrospective study, there were inherent biases and uncontrollable confounding factors. Second, the validation cohort from our center lacked follow-up data to validate prognostic differences concluded by SEER data under different clinicopathological parameters. Finally, the number of patients who were recommended but did not undergo surgery was low, resulting in loss of large amounts of data from the matched cohorts during PSM analysis, which may weaken our conclusions. Therefore, more prospective randomized controlled trials with large samples are warranted to further confirm these findings.

## Conclusion

Generally, PTMC showed higher biological indolence than PTC, which meant higher OS and CSS for patients. For patients with PTMC at stage T1N0M0, AS may be a potentially feasible management strategy. However, the maintenance of good medical compliance and the management of psychological burden should be prioritized for the patients included in AS.

## Data availability statement

The raw data supporting the conclusions of this article will be made available by the authors, without undue reservation.

## Ethics statement

Ethical review and approval was not required for the study on human participants in accordance with the local legislation and institutional requirements. The patients/participants provided their written informed consent to participate in this study.

## Author contributions

Conception and design: XQ and TH; administrative support: XQ, LM, and TH; collection and assembly of data: BQ and JZ; data analysis and interpretation: BQ and LH; manuscript writing: BQ; critical revision of the manuscript: SZ; final approval of manuscript: All authors.

## Acknowledgments

We acknowledge the efforts of the SEER Program tumor registries in providing high quality open resources for researchers.

## Conflict of interest

The authors declare that the research was conducted in the absence of any commercial or financial relationships that could be construed as a potential conflict of interest.

## Publisher’s note

All claims expressed in this article are solely those of the authors and do not necessarily represent those of their affiliated organizations, or those of the publisher, the editors and the reviewers. Any product that may be evaluated in this article, or claim that may be made by its manufacturer, is not guaranteed or endorsed by the publisher.
