# Supplementary material for: Comparison of clinicopathological features and prognosis of papillary thyroid carcinoma and microcarcinoma: A population-based propensity score matching analysis
Source: Front Endocrinol (Lausanne). 2022 Aug 5;13:944758. doi: 10.3389/fendo.2022.944758 (PMC9389084; doi:10.3389/fendo.2022.944758)
Supplement: Supplementary file 1 [file DataSheet_1.docx]

| **Characteristic** | **Level** | **Pre-propensity score matching** | | | | **Post- propensity score matching** | | | |
| --- | --- | --- | --- | --- | --- | --- | --- | --- | --- |
|  |  | **Overall** | **PTC** | **PTMC** | **P** | **Overall** | **PTC** | **PTMC** | **P** |
| N |  | 145951 | 86666 | 59285 |  | 117758 | 58879 | 58879 |  |
| Bone metastases, n (%) | No | 99172 (67.9) | 58718 ( 67.8) | 40454 ( 68.2) | **<0.001** | 80269 (68.2) | 40122 (68.1) | 40147 (68.2) | **<0.001** |
|  | Unknown | 46513 (31.9) | 27712 ( 32.0) | 18801 (31.7) |  | 37309 (31.7) | 18607 (31.6) | 18702 (31.8) |  |
|  | Yes | 266 ( 0.2) | 236 (0.3) | 30 (0.1) |  | 180 (0.2) | 150 (0.3) | 30 (0.1) |  |
| Brain metastases, n (%) | No | 99403 (68.1) | 58924 (68.0) | 40479 (68.3) | **0.005** | 80420 (68.3) | 40248 ( 68.4) | 40172 (68.2) | **0.01** |
|  | Unknown | 46514 (31.9) | 27713 (32.0) | 18801 ( 31.7) |  | 37313 (31.7) | 18611 ( 31.6) | 18702 ( 31.8) |  |
|  | Yes | 34 ( 0.0) | 29 (0.0) | 5 (0.0) |  | 25 ( 0.0) | 20 (0.0) | 5 (0.0) |  |
| Liver metastases, n (%) | No | 99405 (68.1) | 58923 ( 68.0) | 40482 ( 68.3) | **<0.001** | 80418 (68.3) | 40243 ( 68.3) | 40175 ( 68.2) | **<0.001** |
|  | Unknown | 46514 (31.9) | 27713 ( 32.0) | 18801 ( 31.7) |  | 37313 (31.7) | 18611 ( 31.6) | 18702 ( 31.8) |  |
|  | Yes | 32 ( 0.0) | 30 (0.0) | 2 (0.0) |  | 27 (0.0) | 25 (0.0) | 2 (0.0) |  |
| Lung metastases, n (%) | No | 98785 (67.7) | 58345 ( 67.3) | 40440 ( 68.2) | **<0.001** | 79982 (67.9) | 39848 (67.7) | 40134 (68.2) | **<0.001** |
|  | Unknown | 46521 (31.9) | 27718 ( 32.0) | 18803 ( 31.7) |  | 37319 (31.7) | 18615 (31.6) | 18704 (31.8) |  |
|  | Yes | 645 ( 0.4) | 603 (0.7) | 42 (0.1) |  | 457 (0.4) | 416 (0.7) | 41 (0.1) |  |
| Distant LN metastases, n (%) | No | 33408 (22.9) | 20634 ( 23.8) | 12774 ( 21.5) | **<0.001** | 25781 (21.9) | 13048 ( 22.2) | 12733 ( 21.6) | **<0.001** |
|  | Unknown | 112476 (77.1) | 65970 ( 76.1) | 46506 ( 78.4) |  | 91932 (78.1) | 45791 (77.8) | 46141 (78.4) |  |
|  | Yes | 67 ( 0.0) | 62 (0.1) | 5 (0.0) |  | 45 (0.0) | 40 (0.1) | 5 (0.0) |  |
| Tumor size (median [IQR]) |  | 13.0 [7.0, 24.0] | 20.0 [15.0, 31.0] | 5.0 [3.0, 8.0] | **<0.001** | 10.5 [5.0, 20.0] | 20.0 [15.0, 30.0] | 5.0 [3.0, 8.0] | **<0.001** |
| Metastatic LN (median [IQR]) |  | 0.0 [0.0, 2.0] | 1.0 [0.0, 4.0] | 0.0 [0.0, 1.0] | **<0.001** | 0.0 [0.0, 2.0] | 1.0 [0.0, 3.0] | 0.0 [0.0, 1.0] | **<0.001** |
| Follow-up time (median [IQR]) |  | 68.0 [31.0,111.0] | 66.0 [29.0,111.0] | 70.0[34.0,112.0] | **<0.001** | 69.0 [33.0,111.0] | 67.0 [31.0,110.0] | 70.0 [34.0,112.0] | **<0.001** |

Table 1. Supplementary information of the Table 1. Clinicopathological characteristics and statistical results of patients with PTC or PTMC recorded in SEER database before and after propensity score matching.

PTC: papillary thyroid carcinoma; PTMC: papillary thyroid microcarcinoma; N/n: number; LN, lymph node; IQR: interquartile range.

Table 2. Clinicopathological characteristics and statistical results of PTC or PTMC patients who performed surgery and those who were recommended but did not performed surgery recorded in SEER database after propensity score matching.

| **Characteristic** | **Level** | **PTC** | | | | **PTMC** | | | |
| --- | --- | --- | --- | --- | --- | --- | --- | --- | --- |
|  |  | **Overall** | **Performed** | **Recommended** | **P** | **Overall** | **Performed** | **Recommended** | **P** |
| N |  | 1156 | 578 | 578 |  | 238 | 119 | 119 |  |
| Age, (mean (SD)) |  | 54.00 (17.74) | 53.58 (16.90) | 54.41 (18.55) | **0.43** | 51.84 (14.81) | 51.66 (14.19) | 52.02 (15.47) | **0.851** |
| Sex, n (%) | Female | 797 (68.9) | 403 (69.7) | 394 (68.2) | **0.611** | 181 (76.1) | 90 (75.6) | 91 (76.5) | **1** |
|  | Male | 359 (31.1) | 175 (30.3) | 184 (31.8) |  | 57 (23.9) | 29 (24.4) | 28 (23.5) |  |
| Race, n (%) | Black | 88 (7.6) | 45 (7.8) | 43 (7.4) | **0.627** | 12 (5.0) | 5 (4.2) | 7 (5.9) | **0.818** |
|  | Other | 227 (19.6) | 107 (18.5) | 120 (20.8) |  | 58 (24.4) | 30 (25.2) | 28 (23.5) |  |
|  | White | 841 (72.8) | 426 (73.7) | 415 (71.8) |  | 168 (70.6) | 84 (70.6) | 84 (70.6) |  |
| Follicular variant, n(%) | A | 1101 (95.2) | 553 (95.7) | 548 (94.8) | **0.58** | 218 ( 91.6) | 108 (90.8) | 110 (92.4) | **0.815** |
|  | B | 55 (4.8) | 25 (4.3) | 30 (5.2) |  | 20 (8.4) | 11 (9.2) | 9 (7.6) |  |
| Multifocality, n(%) | No | 853 (73.8) | 427 (73.9) | 426 (73.7) | **1** | 173 (72.7) | 87 (73.1) | 86 (72.3) | **1** |
|  | Yes | 303 (26.2) | 151 (26.1) | 152 (26.3) |  | 65 (27.3) | 32 (26.9) | 33 (27.7) |  |
| T stage, n(%) | T1 | 546 (47.2) | 275 (47.6) | 271 (46.9) | **0.27** | 235 (98.7) | 117 (98.3) | 118 (99.2) | **1** |
|  | T2 | 399 (34.5) | 208 (36.0) | 191 (33.0) |  | 0 | 0 | 0 |  |
|  | T3&T4 | 193 (16.7) | 89 (15.4) | 104 (18.0) |  | 3 (1.3) | 2 (1.7) | 1 (0.8) |  |
|  | Tx | 18 (1.6) | 6 (1.0) | 12 (2.1) |  | 0 | 0 | 0 |  |
| N stage, n(%) | N0 | 617 (53.4) | 311 (53.8) | 306 (52.9) | **0.132** | 164 (68.9) | 79 (66.4) | 85 (71.4) | **0.664** |
|  | N1 | 148 (12.8) | 63 (10.9) | 85 (14.7) |  | 21 (8.8) | 12 (10.1) | 9 (7.6) |  |
|  | Nx | 391 (33.8) | 204 (35.3) | 187 (32.4) |  | 53 (22.3) | 28 (23.5) | 25 (21.0) |  |
| M stage, n (%) | M0 | 1053 (91.1) | 532 (92.0) | 521 (90.1) | **0.246** | 230 (96.6) | 116 (97.5) | 114 (95.8) | **0.719** |
|  | M1 | 45 (3.9) | 17 (2.9) | 28 (4.8) |  | 0 | 0 | 0 |  |
| Bone metastases, n (%) | No | 850 (73.5) | 426 (73.7) | 424 (73.4) | **0.958** | 199 (83.6) | 103 (86.6) | 96 (80.7) | **0.293** |
|  | Unknown | 293 (25.3) | 146 (25.3) | 147 (25.4) |  | 39 (16.4) | 16 (13.4) | 23 (19.3) |  |
|  | Yes | 13 (1.1) | 6 (1.0) | 7 (1.2) |  |  |  |  |  |
| Brain metastases, n (%) | No | 861 (74.5) | 431 (74.6) | 430 (74.4) | **0.998** | 199 (83.6) | 103 (86.6) | 96 (80.7) | **0.293** |
|  | Unknown | 293 (25.3) | 146 (25.3) | 147 (25.4) |  | 39 (16.4) | 16 (13.4) | 23 (19.3) |  |
|  | Yes | 2 (0.2) | 1 (0.2) | 1 (0.2) |  |  |  |  |  |
| Liver metastases, n (%) | No | 862 (74.6) | 431 (74.6) | 431 (74.6) | **1** | 199 (83.6) | 103 (86.6) | 96 (80.7) | **0.293** |
|  | Unknown | 294 (25.4) | 147 (25.4) | 147 (25.4) |  | 39 (16.4) | 16 (13.4) | 23 (19.3) |  |
| Lung metastases, n (%) | No | 832 (72.0) | 420 (72.7) | 412 (71.3) | **0.354** | 199 (83.6) | 103 (86.6) | 96 (80.7) | **0.293** |
|  | Unknown | 292 (25.3) | 146 (25.3) | 146 (25.3) |  | 39 (16.4) | 16 (13.4) | 23 (19.3) |  |
|  | Yes | 32 (2.8) | 12 (2.1) | 20 (3.5) |  |  |  |  |  |
| Distant LN metastases, n (%) | No | 441 (38.1) | 232 (40.1) | 209 (36.2) | **0.379** | 55 (23.1) | 30 (25.2) | 25 (21.0) | **0.538** |
|  | Unknown | 713 (61.7) | 345 (59.7) | 368 (63.7) |  | 183 (76.9) | 89 (74.8) | 94 (79.0) |  |
|  | Yes | 2 (0.2) | 1 (0.2) | 1 (0.2) |  |  |  |  |  |
| Tumor size (median [IQR]) |  | 21.0 [15.0, 31.0] | 20.0 [15.0, 30.0] | 21.0 [15.0, 33.0] | **0.062** | 7.0 [5.0, 9.0] | 6.0 [3.5, 8.0] | 9.0 [7.0, 10.0] | **<0.001** |
| Follow-up time (median [IQR]) |  | 36.0 [15.0, 79.0] | 40.0 [19.0, 86.0] | 32.0 [12.0, 69.8] | **<0.001** | 50.0 [23.0, 73.0] | 55.0 [25.5, 75.5] | 46.0 [19.0, 69.5] | **0.096** |
|  | Mx | 58 (5.0) | 29 (5.0) | 29 (5.0) |  | 8 (3.4) | 3 (2.5) | 5 (4.2) |  |
| Cancer-specific death, n(%) | Yes | 39 (3.4) | 12 (2.1) | 27 (4.7) | **<0.001** | 0 | 0 | 0 | **1** |
|  | No | 1117 (96.6) | 566 (97.9) | 551 (95.3) |  | 238 (100.0) | 119 (100.0) | 119 (100.0) |  |
| Status, n (%) | Dead | 171 (14.8) | 57 (9.9) | 114 (19.7) | **<0.001** | 28 (11.8) | 7 (5.9) | 21 (17.6) | **0.009** |
|  | Alive | 985 (85.2) | 521 (90.1) | 464 (80.3) |  | 210 (88.2) | 112 (94.1) | 98 (82.4) |  |

PTC: papillary thyroid carcinoma; PTMC: papillary thyroid microcarcinoma; N/n: number; SD: standard deviation; LN, lymph node; IQR: interquartile range.
